# Exploring novel roles of lipid droplets and lipid metabolism in regulating inflammation and blood–brain barrier function in neurological diseases

**DOI:** 10.3389/fnins.2025.1603292

**Published:** 2025-08-13

**Authors:** Luo Fu, Ting Luo, Zhongnan Hao, Yongli Pan, Wenqiang Xin, Lin Zhang, Zhuhong Lai, Haitao Zhang, Hua Liu, Wei Wei

**Affiliations:** ^1^Department of Neurology, The Affiliated Hospital of Southwest Jiaotong University and The Third People's Hospital of Chengdu, Chengdu, Sichuan, China; ^2^Department of Gastrointestinal and Minimally Invasive Surgery, The Affiliated Hospital of Southwest Jiaotong University and The Third People's Hospital of Chengdu, Chengdu, Sichuan, China; ^3^Department of Ophthalmology, The Affiliated Hospital of Southwest Jiaotong University and The Third People's Hospital of Chengdu, Chengdu, Sichuan, China; ^4^Department of Neurology, University Medical Center of Göttingen, Georg-August-University of Göttingen, Göttingen, Lower Saxony, Germany; ^5^Department of Neurology, Shandong Provincial Hospital Affiliated to Shandong First Medical University, Jinan, Shandong, China; ^6^Jiangxi Key Laboratory of Neurological Diseases, Department of Neurosurgery, The First Affiliated Hospital, Jiangxi Medical College, Nanchang University, Nanchang, Jiangxi, China; ^7^Department of Neurosurgery, Ren Ji Hospital, Shanghai Jiao Tong University School of Medicine, Shanghai, China; ^8^Department of Cardiology, Mianyang Central Hospital, Mianyang, Sichuan, China

**Keywords:** lipid droplets, liposomes, blood–brain barrier, neurological disorders, neuroprotection

## Abstract

The blood–brain barrier (BBB) is a critical structure that maintains the brain’s homeostasis by regulating the transport of molecules and protecting it from harmful substances. However, in neurological diseases such as ischemic stroke, Alzheimer’s disease, Parkinson’s disease, and multiple sclerosis, the integrity and function of the BBB can be significantly compromised. In these conditions, BBB disruption leads to increased permeability, which facilitates neuroinflammation, exacerbates neuronal damage, and accelerates disease progression. Recent research has highlighted the potential of lipid-based carriers, including liposomes and lipid droplets (LDs), in modulating the BBB’s integrity and function in various neurological diseases. Liposomes, with their ability to cross the BBB via mechanisms such as receptor-mediated transcytosis and carrier-mediated transport, are emerging as promising vehicles for the targeted delivery of therapeutic agents to the brain. These properties allow liposomes to effectively reduce infarct size and promote neuroprotection in ischemic stroke, as well as deliver drugs in the treatment of neurodegenerative diseases. Furthermore, LDs—dynamic regulators of lipid metabolism and cellular energy—play an essential role in maintaining cellular homeostasis, particularly during periods of stress when BBB function is compromised. These LDs help sustain cellular energy needs and modulate inflammatory responses, which are key factors in maintaining BBB integrity. Surface modifications of liposomes can further enhance their targeting efficiency, enabling them to selectively bind to specific brain cell types, including neurons, astrocytes, and microglia. This customization improves the precision of therapeutic delivery and supports the development of more tailored treatments. However, challenges such as immune responses, rapid clearance, and complement activation-related toxicity continue to hinder the broader application of liposomes and LDs in clinical settings. This review will focus on the roles of liposomes and LDs in regulating BBB integrity across a range of neurological diseases, discussing their potential for targeted drug delivery, neuroprotection, and the modulation of neuroinflammation. Additionally, we will explore the strategies being developed to address the limitations that currently restrict their clinical use.

## 1 Introduction

The blood–brain barrier (BBB) is a dynamic and highly selective interface that separates the circulating blood from the brain parenchyma (Keaney and Campbell, 2015). Composed primarily of endothelial cells, tight junction proteins, astrocytes, and pericytes, the BBB plays a crucial role in maintaining central nervous system (CNS) homeostasis by regulating the transport of ions, nutrients, signaling molecules, and lipids (Correale and Villa, 2009; Singh and Vellapandian, 2023; Lochhead et al., 2020). Among the critical lipid species required for brain function is docosahexaenoic acid (DHA), an omega-3 fatty acid essential for neuronal development and synaptic function (Dyall, 2015; Sinclair, 2019). The transport of DHA across the BBB is largely mediated by the lipid transporter MFSD2A, which highlights the specialized lipid transport mechanisms necessary to support brain metabolism and function (Chan et al., 2018; Wood et al., 2021). Lipid metabolism is increasingly recognized as a fundamental component of both physiological BBB maintenance and pathological disruption (Pifferi et al., 2021). In this context, lipid droplets (LDs), intracellular organelles involved in lipid storage, signaling, and energy homeostasis, have emerged as key regulators of cellular stress responses, including inflammation and oxidative stress, particularly in neural and endothelial cells (Geltinger et al., 2020; Zhang et al., 2025). Under pathological conditions, the dynamics of LDs are closely associated with neuroinflammatory responses, mitochondrial dysfunction, and the integrity of the endothelial barrier (Parodi-Rullán et al., 2021; Chen et al., 2024; Zhong et al., 2025). For instance, during ischemic stroke, energy failure and oxidative stress compromise the structural and functional integrity of the BBB, resulting in increased permeability, vasogenic edema, and infiltration of peripheral immune cells and neurotoxic substances (Fang et al., 2024; Bernardo-Castro et al., 2020). This BBB breakdown exacerbates neuronal injury and promotes a harmful neuroinflammatory cascade (Gao et al., 2023). In parallel, the development of lipid-based therapeutic strategies—most notably liposomes and LD-modulating agents—has gained traction for their potential in restoring BBB integrity and enhancing drug delivery to the brain (Juhairiyah and de Lange, 2021; Mondal and Ghosh, 2023). Liposomes, due to their amphiphilic bilayer structure, can encapsulate both hydrophilic and hydrophobic drugs, protect them from enzymatic degradation, and facilitate targeted transport across the BBB (Sonju et al., 2021; Bruch et al., 2019). Meanwhile, LDs are increasingly studied not only for their involvement in cellular stress regulation but also as potential targets for modulating inflammation and metabolic dysregulation during cerebrovascular injury (Tan et al., 2024; Lan et al., 2023).

## 2 Lipid metabolism and neurological disorders

### 2.1 The brief course on lipid metabolism

Lipid metabolism encompasses the complex biochemical processes involved in the synthesis, transport, and degradation of lipids (Ridgway and McLeod, 2008). These processes are crucial for maintaining cellular homeostasis, energy production, and structural integrity of biological membranes (Fagone and Jackowski, 2009). The major pathways of lipid metabolism include fatty acid oxidation, lipid synthesis, and lipoprotein metabolism (Rinaldo et al., 2002). Dysregulation of lipid metabolism has been implicated in various pathological conditions, including metabolic syndrome, cardiovascular diseases, and neurological disorders (van Meer et al., 2008). In the nervous system, lipids play fundamental roles in neuronal function, including signal transduction, membrane fluidity, and myelination (Dietschy and Turley, 2004). The brain, despite comprising only about 2% of body weight, contains nearly 50% of the body’s total lipids, highlighting the significance of lipid metabolism in neural health and disease (Sonnino et al., 2015).

### 2.2 Biogenesis of LDs

**Figure 1:**
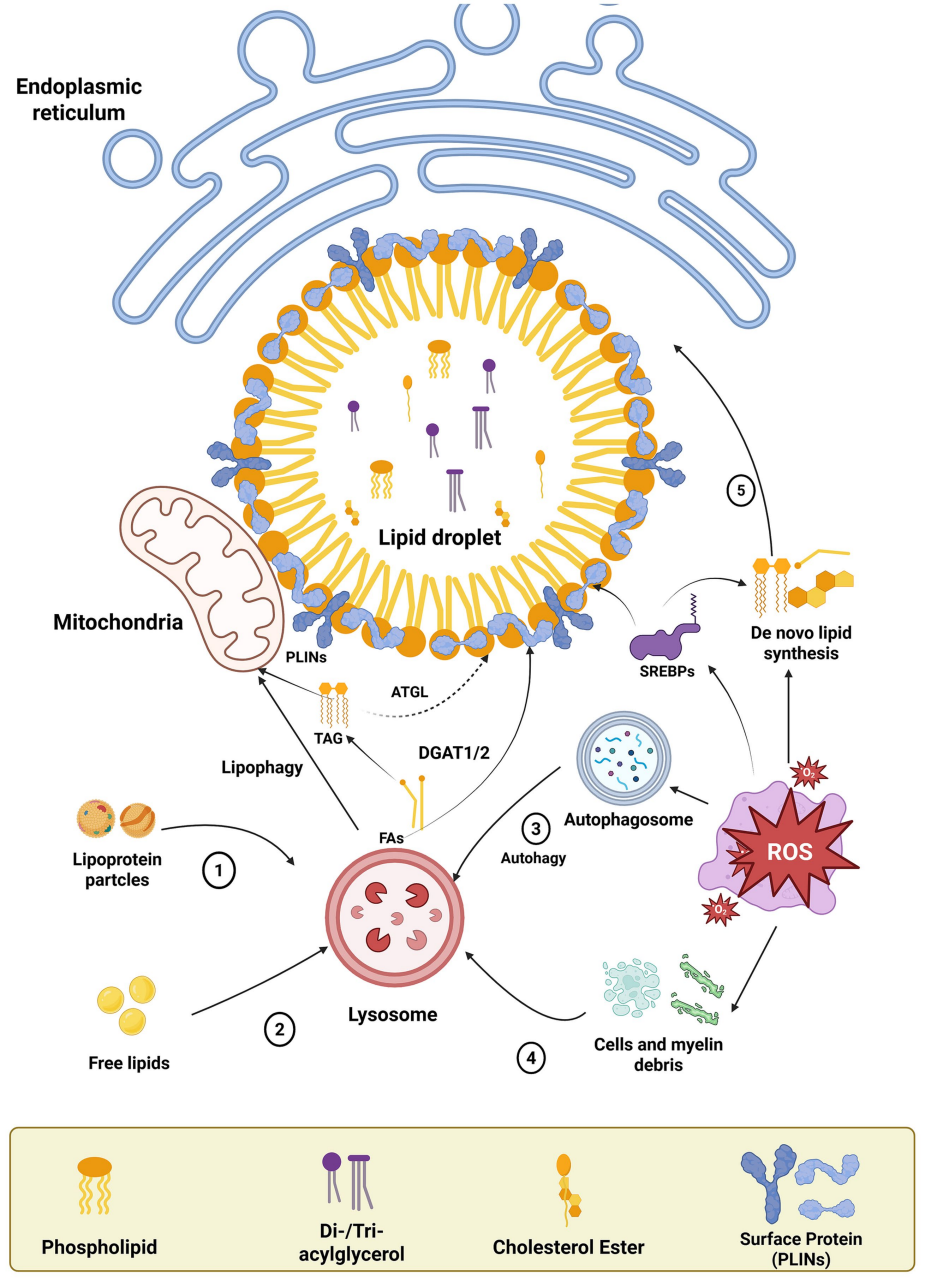
Lipid droplet biogenesis. Lipid droplets (LDs) are spherical organelles with a unique structure consisting of a core of neutral lipids, mainly triglycerides (TG) and cholesteryl esters. Their core is surrounded by a monolayer of phospholipids and associated proteins. LDs are dynamic and functionally active organelles involved in various functions such as lipid metabolism, cell signaling and inflammation.

LDs are dynamic organelles that store neutral lipids, primarily triglycerides and cholesteryl esters (Walther and Farese, 2012). They are formed through the coordinated actions of the endoplasmic reticulum (ER) and various lipid metabolism enzymes (Olzmann and Carvalho, 2019). LD biogenesis is initiated by the deposition of neutral lipids within the ER membrane bilayer, which subsequently buds outward to generate mature lipid droplets (Thiam and Beller, 2017) (Figure 1). LDs were once considered inert lipid reservoirs, but recent studies reveal their active role in lipid homeostasis, signaling, and cellular stress responses (Wang, 2016). In neurological contexts, LDs are increasingly recognized as key regulators of neuronal lipid metabolism, oxidative stress mitigation, and neuroinflammation (Farmer et al., 2020).

### 2.3 The vital role of lipid metabolism in neurological disorders

**Figure 2:**
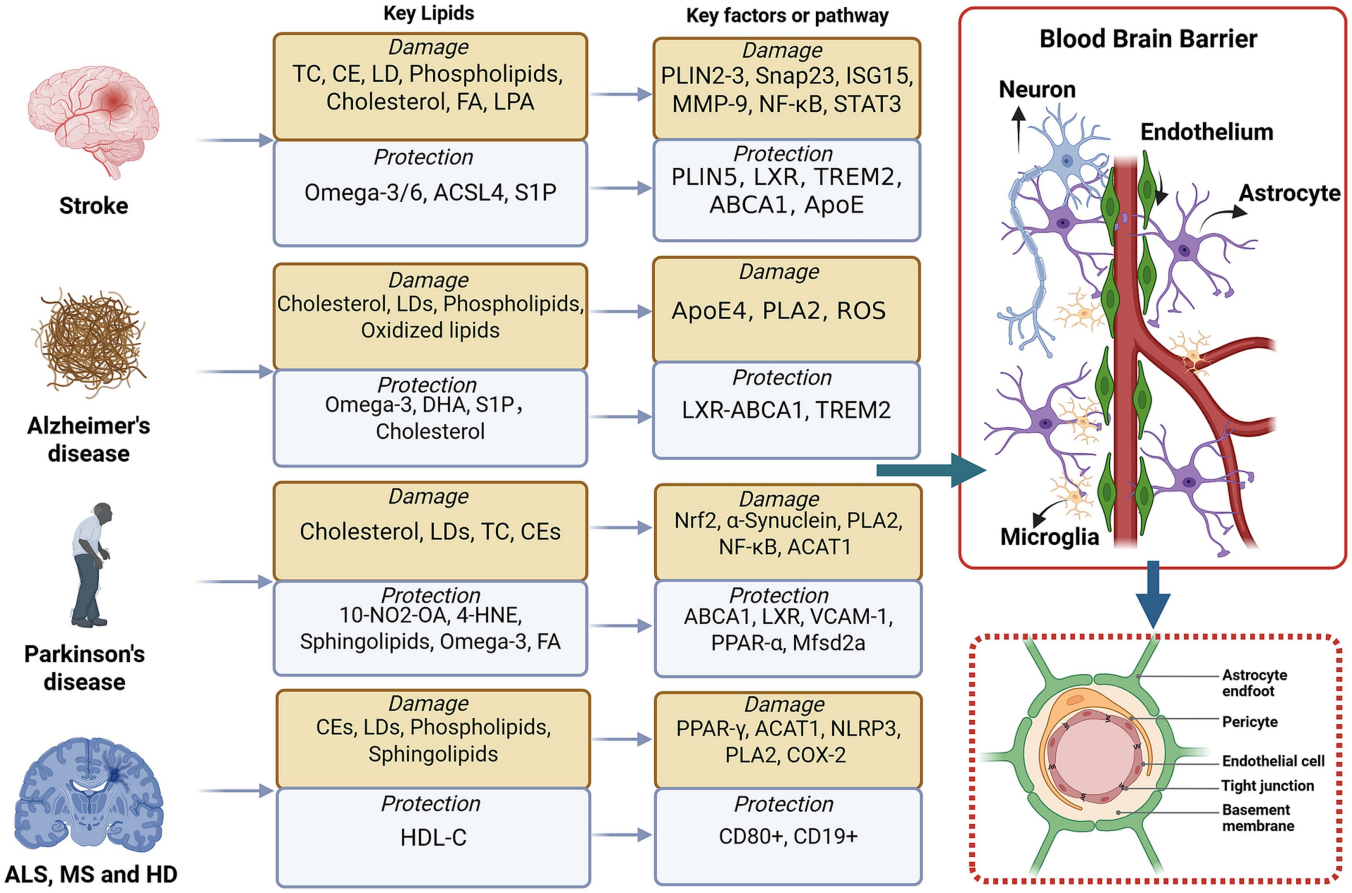
Schematic illustration of lipids and their respective roles in neurological diseases. In the central nervous system, lipid metabolism and lipid LDs play a crucial role in modulating neuroinflammation and preserving BBB integrity in neurological disorders. Disruptions in lipid homeostasis can impair the function of key cellular components, including neurons, microglia, astrocytes, and endothelial cells. Through the regulation of lipid metabolism, processes such as chronic inflammation, neuronal injury, and BBB dysfunction can be influenced, thereby impacting the progression and potential recovery of central nervous system disorders, including Stroke, Alzheimer’s disease (AD), Parkinson’s disease (PD), amyotrophic lateral sclerosis (ALS), Huntington’s disease (HD), and multiple sclerosis (MS). BBB, Blood–brain barrier; AS, Astrocytes; EC, Endothelial cell; CEs, Cholesterol esters; TC, Triglycerides; FA, Fatty Acid; LDs, Lipid droplets; LPA, Lysophosphatidic acid; ROS, Reactive oxygen species; ICH, Intracerebral hemorrhagic stroke; IS, Ischemic stroke; ACSL4, Acyl-CoA synthetase long-chain family member 4; S1P, Sphingosine-1-phosphate; S1PR3, Sphingosine-1-phosphate receptor 3; AD, Alzheimer’s disease; TREM2, Triggering Receptor Expressed on Myeloid cells 2; ApoE, Apolipoprotein E; LXR, Liver X Receptor; ABCA1, ATP-binding cassette transporter A1; PLA2, Phospholipase A2; PD, Parkinson’s disease; COX-2, Cyclooxygenase-2; PPAR-α, Peroxisome proliferator-activated receptor alpha; ACAT1, Acyl-CoA cholesterol acyltransferase 1; 4-HNE, 4-Hydroxynonenal; 10-NO2-OA, Nitroalkene 10-nitro-oleic acid; MS, Multiple sclerosis; HD, Huntington’s disease; ALS, Amyotrophic lateral sclerosis; HDL-C, high-density lipoprotein cholesterol.

Lipid metabolism is intricately involved in maintaining neuronal function, membrane integrity, and energy homeostasis, and its dysregulation has been implicated in a wide range of neurological disorders (Tracey et al., 2018; Zhao et al., 2023) (Figure 2). In Alzheimer’s disease (AD), impaired lipid metabolism contributes to Aβ aggregation and tau pathology, with cholesterol modulating APP processing and ApoE4 impairing Aβ clearance (Rajmohan and Reddy, 2017). Lipid peroxidation products further amplify oxidative stress and neuroinflammation (Maulik et al., 2013). Similarly, in Parkinson’s disease (PD), especially peroxidized PUFAs and disrupted sphingolipids, promotes α-synuclein aggregation, mitochondrial dysfunction, and lysosomal impairment (Mesbahi, 2023). In multiple sclerosis (MS), disturbed lipid homeostasis affects myelin synthesis and oligodendrocyte function, while lipid mediators such as eicosanoids and sphingolipids modulate immune responses and neuroinflammation (López-Muguruza and Matute, 2023). In Huntington’s disease (HD), involved defective cholesterol and phospholipid metabolism due to mutant huntingtin-induced SREBP dysregulation, compromising synaptic function and neuronal viability (Leoni and Caccia, 2015). In amyotrophic lateral sclerosis (ALS), abnormal fatty acid utilization, hypermetabolism, and dyslipidemia contribute to motor neuron degeneration and inflammation (D'Amico et al., 2021). In ischemic stroke (IS), lipid metabolism plays dual roles in neuroprotection and neurotoxicity by regulating inflammation, oxidative stress, and BBB integrity through phospholipid and sphingolipid pathways (Sun et al., 2016; Zhao et al., 2022; Sandoval and Witt, 2008), whose disruption exacerbates vascular permeability, brain edema, and neuronal injury (Sandoval and Witt, 2008; Arbaizar-Rovirosa et al., 2023). Lipid peroxidation, triggered by oxidative stress following ischemia–reperfusion injury, generates reactive oxygen species (ROS) that further damage neuronal membranes and promote inflammation (Wu et al., 2020). Moreover, cholesterol metabolism influences post-stroke recovery, with evidence suggesting that high levels of oxidized cholesterol derivatives contribute to neuroinflammatory cascades (Ciancarelli et al., 2022; Kloska et al., 2020). Collectively, these findings highlight the fundamental role of lipid metabolism in neurological disorders, emphasizing its impact on neurodegeneration, neuroinflammation, cerebrovascular dysfunction, and bioenergetic deficits (Yin, 2023; Helgudóttir et al., 2024; Clemente-Suárez et al., 2024). Understanding these mechanisms may provide novel therapeutic targets aimed at modulating lipid metabolism to protect against neuronal dysfunction, enhance neuroprotection, and improve recovery following acute neurological insults.

### 2.4 Lipid metabolism in BBB penetration

Lipid metabolism plays a fundamental role in maintaining the structural and functional integrity of the BBB, a highly selective barrier that regulates molecular exchange between the bloodstream and the CNS (Pifferi et al., 2021). The BBB is composed of endothelial cells with tight junctions, pericytes, and astrocyte end-feet, all of which rely on tightly regulated lipid homeostasis to preserve barrier integrity and control permeability (Singh and Vellapandian, 2023). The lipid composition of the BBB is unique, being highly enriched in sphingolipids, phospholipids, and cholesterol, which are critical for maintaining membrane fluidity, vesicular trafficking, and signaling (Wang et al., 2020). Changes in lipid metabolism can directly influence BBB function, either enhancing its protective role or contributing to its dysfunction in neurological diseases. For instance, sphingolipids and ceramides are known regulators of BBB permeability, with their dysregulation leading to increased endothelial cell apoptosis, inflammation, and barrier disruption (Kuperberg and Wadgaonkar, 2017). Cholesterol metabolism also plays a crucial role, as cholesterol is a key component of endothelial membranes and lipid rafts, which mediate signal transduction and transport mechanisms at the BBB (Dai et al., 2021). However, excessive cholesterol accumulation or the presence of oxidized cholesterol derivatives can trigger inflammatory cascades, leading to endothelial dysfunction and increased BBB permeability, which has been implicated in diseases such as AD, MS, and stroke (Candore et al., 2010).

Lipid transporters and lipid-binding proteins further influence BBB penetration and function (Yamazaki et al., 2019). Apolipoproteins such as ApoE, which facilitates lipid transport in the brain via lipoprotein particles, significantly impact BBB dynamics. The ApoE4 variant, for example, has been associated with BBB breakdown and neurovascular dysfunction, increasing the risk of neurodegenerative diseases (Montagne et al., 2020). Additionally, ATP-binding cassette transporters, such as ABCA1 and ABCG1, regulate cholesterol and lipid efflux from endothelial cells, thereby modulating BBB permeability (Rohrer et al., 2009). Dysregulation of these transporters can impair lipid clearance, leading to increased neuroinflammation and neurovascular damage. Fatty acid metabolism also plays a critical role in BBB function, as endothelial cells utilize specific fatty acid oxidation pathways to maintain barrier homeostasis (Fock and Parnova, 2023). Disruptions in fatty acid oxidation have been linked to increased BBB permeability and neuroinflammatory responses, particularly in conditions like MS and ischemic stroke (Mitchell and Hatch, 2011).

Beyond its endogenous functions, lipid metabolism is also crucial for drug delivery strategies targeting the CNS (Correia et al., 2022). The BBB presents a major challenge for therapeutic agents due to its restrictive permeability, but lipid-based approaches have been explored to enhance drug transport across the barrier (Wu et al., 2023). Although lipophilic drugs, lipid nanoparticles, and liposome-based carriers have been explored to enhance drug delivery across the BBB via lipid-mediated transport, the notion that liposomes easily cross the BBB is often misleading (Micheli et al., 2012). While PEGylation improves systemic circulation by reducing clearance, it does not inherently facilitate BBB penetration and may even hinder interaction with the endothelial surface. Effective transport across the BBB typically requires active targeting strategies, such as receptor-mediated transcytosis. This involves functionalizing liposomes with ligands or peptides that bind to specific receptors expressed on brain endothelial cells—such as the transferrin receptor (TfR), insulin receptor (IR), and low-density lipoprotein receptor (LDLR). These receptors naturally mediate the transport of essential molecules like iron, insulin, and cholesterol into the brain. By mimicking endogenous ligands, targeted liposomes can hijack these pathways to enhance BBB penetration. However, such strategies are still underutilized and often insufficiently optimized in preclinical models, limiting their translational impact. Nevertheless, understanding the interplay between lipid metabolism and BBB function not only provides insights into disease mechanisms but also opens avenues for developing novel therapeutics aimed at restoring BBB integrity and enhancing drug delivery for neurological disorders.

## 3 Lipid metabolism and inflammation

### 3.1 The brief course on inflammation

Inflammation plays a central role in neurological diseases, acting as both an acute defense mechanism and a contributor to chronic neurodegeneration when dysregulated (Leng and Edison, 2021; Marogianni et al., 2020; Tansey et al., 2022). In the CNS, inflammation is primarily mediated by microglia and astrocytes, which become activated in response to injury or pathological stimuli, releasing pro-inflammatory cytokines such as TNF-α, IL-1β, and IL-6, as well as ROS, leading to neuronal damage and BBB disruption (Sochocka et al., 2017). In AD, Aβ deposition persistently stimulates microglia, promoting chronic neuroinflammation that accelerates neuronal degeneration (Leng and Edison, 2021). In PD, aggregated α-synuclein triggers microglial activation, releasing toxic mediators that exacerbate dopaminergic neuron loss (Zhang et al., 2005). MS is characterized by T-cell-mediated autoimmune attacks on myelin, leading to demyelination, neurodegeneration, and lipid metabolism dysregulation, further impairing myelin synthesis and repair (Correale et al., 2019). In ischemic stroke, hypoxia-ischemia induces an inflammatory cascade where activated microglia and infiltrating peripheral immune cells release inflammatory mediators, amplifying neuronal injury, while lipid peroxidation and BBB breakdown further exacerbate inflammation (Zhang, 2014; Maida et al., 2020). Additionally, lipid metabolism plays a crucial role in regulating neuroinflammation, with arachidonic acid-derived prostaglandins and leukotrienes promoting inflammation, whereas ω-3 fatty acid metabolites such as resolvins and protecting facilitate inflammation resolution and homeostasis (Shang et al., 2019; Farooqui, 2012). The intricate crosstalk between inflammation and lipid metabolism is particularly evident in neurodegenerative diseases, cerebrovascular disorders, and neuroimmune conditions, highlighting potential therapeutic targets for anti-inflammatory and metabolic interventions.

### 3.2 A double-edged sword role of lipids and possible signal pathway

Lipid metabolism plays a paradoxical role in the CNS, acting as both a protector of homeostasis and a contributor to disease progression (Zhang et al., 2025; Walther and Farese, 2012). On one hand, lipids are essential for neuronal membrane integrity, synaptic function, and energy metabolism (Walther and Farese, 2012). Dysregulated lipid pathways can drive neuroinflammation, oxidative stress, and neurodegeneration (Zhang et al., 2025; Farmer et al., 2020). Several key lipid-related signaling pathways illustrate this dual role in neurological health and disease. The SREBP pathway, a central regulator of cholesterol and lipid homeostasis, is vital for maintaining neuronal function (Shimano and Sato, 2017). Under normal conditions, SREBP activation ensures adequate lipid supply for membrane synthesis and repair (Wang et al., 2025). However, in diseases such as AD and HD, impaired SREBP signaling disrupts lipid balance, leading to synaptic dysfunction and neuronal death (Wang et al., 2025; Hu et al., 2023; Li et al., 2020). Similarly, the PPAR (Peroxisome Proliferator-Activated Receptor) family, particularly PPAR-γ, plays a neuroprotective role by regulating fatty acid oxidation, anti-inflammatory responses, and mitochondrial function (Kapadia et al., 2008; Fidaleo et al., 2014; Escandon et al., 2021). Activation of PPAR-γ has been shown to mitigate neuroinflammation in PD and MS (Escandon et al., 2021; Chen et al., 2012). However, excessive PPAR-γ activation may also lead to lipid accumulation, contributing to metabolic stress and neuronal vulnerability (Xi et al., 2020). Sphingolipid metabolism exemplifies the complex interplay between lipid signaling, neuroinflammation, and BBB regulation (Arsenault et al., 2021). A central mediator in this pathway is sphingosine-1-phosphate (S1P), a bioactive lipid that binds to a family of G-protein–coupled receptors (S1PR1–S1PR5) expressed on endothelial cells, astrocytes, and immune cells. S1P signaling plays a dual role: it promotes BBB stability by enhancing endothelial cell junction integrity and simultaneously regulates immune cell trafficking across the BBB (Cartier and Hla, 2019; Wang et al., 2025). Dysregulation of this pathway contributes to pathological immune infiltration and barrier breakdown in neuroinflammatory disorders (Wang et al., 2025). Therapeutically, S1P receptor modulators such as fingolimod (FTY720) function by downregulating S1PR1 on lymphocytes, thereby sequestering them in lymph nodes and reducing CNS infiltration (Brinkmann, 2009; Pournajaf et al., 2022). In MS, this mechanism attenuates neuroinflammation and protects BBB integrity, making S1P signaling a critical target for both immune modulation and vascular stabilization (Bravo et al., 2022; Groves et al., 2013). However, elevated levels of ceramides and sphingolipids can induce apoptosis and neurotoxicity, exacerbating disease pathology in stroke and neurodegenerative disorders (Farooqui and Farooqui, 2024).

LDs, once considered passive fat reservoirs, have emerged as critical regulators of cellular homeostasis (Tracey et al., 2018). In glial cells, LDs sequester toxic lipid peroxidation products, protecting neurons from oxidative damage (Olzmann and Carvalho, 2019). Persistent accumulation of LDs under pathological conditions—such as in ALS, PD, and other neuroinflammatory disorders—has been increasingly recognized as a contributor to chronic inflammation and metabolic dysfunction (Farmer et al., 2020). LDs serve not only as energy reserves but also as platforms for the synthesis and storage of bioactive lipids, including pro-inflammatory mediators (Tan et al., 2024; Zhang et al., 2025). A key inflammatory pathway linked to LD metabolism is the cyclooxygenase-lipoxygenase (COX-LOX) axis, which metabolizes arachidonic acid into a diverse range of eicosanoids, such as prostaglandins, leukotrienes, and thromboxanes (Wang et al., 2021; Rudrapal et al., 2023). These lipid mediators orchestrate immune cell recruitment, glial activation, oxidative stress, and vascular permeability—processes that are central to the progression of neurodegenerative diseases (Rudrapal et al., 2023; Adibhatla and Hatcher, 2010). In PD, aberrant activation of the COX-2 isoform has been implicated in dopaminergic neuron loss, with elevated levels of PGE2 detected in affected brain regions (Kumar et al., 2020). While PGE2 and leukotrienes amplify inflammatory responses in acute CNS injury and chronic neurodegeneration, their counterbalancing counterparts, such as resolvins and protectins, derived from omega-3 fatty acids, promote the resolution of inflammation and neuroprotection (Artru et al., 2022). Similarly, LOX-derived leukotrienes have been associated with microglial activation and neurotoxicity (Aoki et al., 2023). However, evidence remains conflicted, as some lipid mediators—such as lipoxins and resolvins—produced downstream of LOX pathways exhibit anti-inflammatory and pro-resolving effects (Spite et al., 2014; Buckley et al., 2014). This dual nature highlights the complexity of targeting the COX-LOX axis therapeutically: while inhibition of COX-2 has shown neuroprotective effects in preclinical PD models, clinical outcomes with COX inhibitors have been inconsistent, possibly due to interference with beneficial lipid mediators or compensatory pathway activation (Mukhopadhyay et al., 2023; Agrawal, 2025). Adding to this complexity is the role of acyl-CoA cholesterol acyltransferase 1 (ACAT1), an enzyme that regulates intracellular cholesterol esterification and LD formation (Xu et al., 2019; Chang et al., 2001). In PD, ACAT1 has been found to be upregulated in activated microglia and is associated with cholesterol ester accumulation and sustained neuroinflammatory responses (Chang et al., 2001; Huynh et al., 2024). Genetic or pharmacological inhibition of ACAT1 has been shown to reduce LD burden, suppress pro-inflammatory cytokine release, and protect dopaminergic neurons in experimental models of PD and AD (Huynh et al., 2024; Sun et al., 2025; Valencia-Olvera et al., 2023). These findings suggest that targeting ACAT1 may not only restore lipid homeostasis but also indirectly modulate COX-LOX signaling by limiting the availability of lipid substrates stored in LDs.

These observations underscore the central role of lipid-mediated inflammation in neurodegeneration, while also revealing the intricate balance between protective and harmful lipid signaling. The imbalance between pro-inflammatory and pro-resolving lipid mediators is a hallmark of neuroinflammatory diseases (Ahluwalia et al., 2022). These findings highlight the intricate role of lipid metabolism in neurological disorders, where the same pathways that sustain brain homeostasis can, under pathological conditions, drive disease progression. Understanding these signaling networks may pave the way for targeted lipid-based therapies that balance neuroprotection and metabolic regulation in CNS disorders.

### 3.3 The crosstalk of impact of inflammation on LDs

Emerging evidence suggests that LDs function as key regulators of cellular responses to stress, particularly in the context of inflammation (Walther and Farese, 2012). The interplay between inflammation and LD metabolism is bidirectional: inflammatory stimuli can induce LD biogenesis and alter lipid composition, while LDs, in turn, modulate inflammatory signaling pathways by sequestering bioactive lipids and influencing immune cell function (Tan et al., 2024; Monson et al., 2021). Inflammation profoundly impacts LD formation and turnover through multiple mechanisms. Pro-inflammatory cytokines such as TNF-α, IL-1β, and IFN-γ promote LD accumulation in various cell types, including microglia, astrocytes, and endothelial cells (Rodríguez-Gómez et al., 2020; Fornari Laurindo et al., 2023). This process serves both protective and pathological roles—on one hand, LDs act as reservoirs to store potentially harmful lipids, preventing lipotoxicity and excessive oxidative stress (Geltinger et al., 2020; Pressly et al., 2022). On the other hand, LDs serve as platforms for the synthesis of eicosanoids, prostaglandins, and leukotrienes, which are lipid mediators that amplify the inflammatory response (Pereira-Dutra and Bozza, 2021). Additionally, chronic inflammation disrupts LD homeostasis, leading to excessive lipid accumulation, mitochondrial dysfunction, and sustained immune activation, all of which contribute to neuroinflammation and disease progression (Leyane et al., 2022).

The regulation of LD dynamics during inflammation is tightly controlled by lipid metabolism-associated transcription factors, transporters, and structural proteins (Florance and Ramasubbu, 2022). Several key regulators, including TREM2, SREBPs, ABCA1, Perilipins (PLIN2-5), ApoE, and LXRs, play crucial roles in balancing LD formation, lipid transport, and inflammatory signaling (Florance and Ramasubbu, 2022). Understanding how these factors mediate the crosstalk between inflammation and LDs provides important insights into potential therapeutic targets for neuroinflammatory and neurodegenerative diseases.

### 3.4 Key regulatory factors in the crosstalk between inflammation and LDs

#### 3.4.1 TREM2

TREM2 is a lipid-sensing receptor expressed in microglia that plays a critical role in lipid uptake, LD biogenesis, and the resolution of inflammation (Hou et al., 2022). Under inflammatory conditions, TREM2 facilitates the clearance of apoptotic cells and lipid debris by promoting phagocytosis and lipid catabolism. Loss-of-function mutations in TREM2, such as those associated with neurodegenerative diseases, impair LD metabolism, leading to lipid overload, increased oxidative stress, and chronic microglial activation (Klionsky et al., 2021; Smolič et al., 2021). Furthermore, TREM2-deficient microglia fail to efficiently regulate inflammation, resulting in prolonged cytokine release and exacerbated neurodegeneration (Hou et al., 2022). The interaction between TREM2 and LDs highlights the importance of lipid metabolism in controlling inflammatory responses in the brain (Gouna, 2024).

#### 3.4.2 SREBPs

SREBPs are master regulators of lipid biosynthesis, controlling the expression of genes involved in fatty acid and cholesterol synthesis (Eberlé et al., 2004). Inflammation activates SREBPs through cytokine signaling pathways, leading to increased lipid accumulation within LDs (Sahini and Borlak, 2014). While SREBP activation is essential for maintaining membrane integrity and cellular lipid reserves, excessive activation in response to chronic inflammation can result in lipid imbalances, mitochondrial dysfunction, and inflammatory stress (Jarc and Petan, 2019). In microglia and astrocytes, SREBP-driven lipid accumulation contributes to a pro-inflammatory state by fueling the production of prostaglandins and other eicosanoids (Zhang et al., 2025). Additionally, cross-talk between SREBPs and inflammatory pathways, such as NF-κB signaling, further amplifies immune activation, linking lipid dysregulation to neuroinflammatory disorders (Xu et al., 2024; Li et al., 2022).

#### 3.4.3 ABCA1

ABCA1 is a key lipid transporter responsible for cholesterol and phospholipid efflux, playing a vital role in LD homeostasis and inflammatory regulation (Kotlyarov, 2021). Inflammatory stimuli downregulate ABCA1 expression, leading to impaired lipid clearance, increased LD accumulation, and exacerbated immune activation (Wang and Westerterp, 2020). In contrast, enhancing ABCA1 function promotes lipid efflux, reducing inflammatory lipid species and protecting against neuroinflammatory damage (Karasinska et al., 2013). ABCA1 also influences microglial polarization by modulating the balance between pro-inflammatory M1 and anti-inflammatory M2 phenotypes, demonstrating its role in lipid-mediated immune regulation (Reid, 2023; Wei et al., 2024).

#### 3.4.4 PLINs

Perilipins (PLINs) are structural proteins that coat LDs and regulate lipid storage, hydrolysis, and signaling (Chandrasekaran et al., 2024). Among them, PLIN2 and PLIN3 are highly induced during inflammation, promoting LD expansion and stabilizing lipid storage under oxidative stress (Chandrasekaran et al., 2024; Griseti et al., 2024). PLIN5, in contrast, plays a role in mitochondrial-lipid interactions, facilitating lipid utilization to prevent excessive accumulation (Dabravolski et al., 2021). Dysregulation of PLIN proteins during chronic inflammation leads to lipid overload, increased ROS production, and heightened immune activation (Bombarda-Rocha et al., 2023). Studies suggest that targeting PLIN-regulated LD dynamics may help modulate inflammation in neurodegenerative conditions.

#### 3.4.5 ApoE

ApoE is a critical lipid transport protein in the brain that influences LD metabolism and inflammation (Husain et al., 2021). ApoE facilitates cholesterol and phospholipid transport between cells, regulating lipid availability for LD biogenesis (Hauser et al., 2011). However, ApoE4, a genetic variant linked to neurodegenerative diseases, is associated with altered lipid metabolism, impaired LD function, and heightened inflammatory responses. ApoE deficiency or dysfunction exacerbates neuroinflammation by promoting lipid accumulation, reducing lipid clearance efficiency, and amplifying microglial activation (Montagne et al., 2020; Husain et al., 2021; Ayyubova, 2024). These findings highlight ApoE’s essential role in balancing lipid metabolism and immune regulation in the CNS.

#### 3.4.6 LXRs

LXRs are nuclear receptors that regulate cholesterol homeostasis, lipid transport, and inflammatory gene expression (Wang and Tontonoz, 2018). LXRs promote ABCA1-mediated lipid efflux, reducing LD accumulation and mitigating inflammatory stress (Gu et al., 2019; Wu et al., 2019). Additionally, LXRs suppress pro-inflammatory cytokine production through direct transcriptional repression of NF-κB target genes, establishing a protective mechanism against excessive immune activation (A-González and Castrillo, 2011; Zhao et al., 2021). Pharmacological activation of LXRs has been proposed as a therapeutic strategy to enhance lipid metabolism and counteract inflammation in neurodegenerative diseases (Xu et al., 2013; Fitz et al., 2019). However, the precise role of LXRs in different CNS cell types remains an area of active investigation.

## 4 Lipid metabolism is involved in regulating inflammation in neurological disorders

### 4.1 Stroke

Stroke, including ischemic and hemorrhagic subtypes, triggers a complex cascade of inflammatory responses that significantly impact neuronal survival, brain repair, and long-term recovery (Reid, 2023). In stroke, particularly ischemic stroke, lipid metabolism exerts a dual role in neuroprotection and neurotoxicity by modulating inflammatory responses (Xu et al., 2021), immune cell activation, oxidative stress, and maintaining BBB integrity through phospholipid and sphingolipid pathways (Sun et al., 2016; Zhao et al., 2022; Sandoval and Witt, 2008), whose disruption exacerbates vascular permeability, brain edema, and neuronal injury (Sandoval and Witt, 2008; Arbaizar-Rovirosa et al., 2023). Dysregulated lipid metabolism in stroke can either exacerbate neuroinflammation and BBB disruption or facilitate resolution of inflammation and neuroprotection, making it a crucial factor in stroke pathophysiology (Zhang et al., 2025; Bernardo-Castro et al., 2020; Yang et al., 2019; Jiang et al., 2021; Janssen et al., 2021; Cui et al., 2021) (Table 1).

**Table 1:** Preclinical studies assessing the effect of lipids on neuroinflammation or BBB function in stroke.

References	Country	Species	Type of stroke	Involved cells	Damage or protection	Key lipids	Key factors or pathways	Effects on BBB	Mechanisms
Wei et al. (2024)	Germany	Mice, Cells	IS	Microglia, AS	Damage	TC, CE, LDs	ABCA1, ApoE	Inflammation	LDs accumulation drives microglial inflammation and dysfunction, exacerbating post-stroke neuroinflammation
Arbaizar-Rovirosa et al. (2023)	Spain	Mice, Human	IS	Microglia	NA	Phospholipids, Cholesterol	PLIN1-5, Snap23, ISG15	Phagocytosis, Inflammation	Accumulated LDs exaggerated inflammatory responses after stroke
Haley et al. (2020)	UK	Mice	IS	Neuron	NA	FA, TC	NA	Inflammation	Stroke causes lasting metabolic, hepatic, and behavioral disturbances.
Janssen et al. (2021)	Germany	Mice, Cells	IS	AS, EC	Protection	FA	ABCB1, MMP-9, NF-κB	Inflammation, Apoptosis	FAS inhibition worsens stroke injury by promoting BBB breakdown and inflammation.
Ament et al. (2024)	USA	Mice, Human	IS	Neuron	Protection	Omega-3	NA	Inflammation	Plasma DHA lipids reduce ischemic stroke risk and mediate fish intake benefits
Lei et al. (2025)	China	Mice, Cells	IS	Microglia	NA	LDs	LXR, ABCA1, TLR4	Inflammation	CKN alleviates ischemic stroke injury via LXRα/ABCA1 activation and TLR4 inhibition.
Zeng et al. (2020)	China	Rats	IS	Neuron	NA	LPA	LXR, NF-κB	Inflammation	LPA promotes ischemic injury via NFκB activation and LXR suppression.
Wu et al. (2024)	China	Mice, Cells	ICH	OPCs	NA	LDs	DLK1/AMPK/ACC, STAT3	ROS, Ferroptosis	IL-10 protects OPCs post-stroke by reducing lipid ROS and ferroptosis.
Cui et al. (2021)	China	Mice	IS	Neuron	NA	ACSL4	HIF-1α	Ferroptosis, Inflammation	ACSL4 promotes ischemic stroke injury by enhancing lipid peroxidation and neuroinflammation, making it a potential therapeutic target.
Wei et al. (2024)	Germany	Mice, Cells	IS	Microglia	NA	FA, TC, CE, LDs	TREM2, PLIN2, ApoE, ABCA1	Inflammation	TREM2 regulates microglial lipid metabolism, reducing stroke inflammation.
Fan et al. (2022)	China	Mice	IS	NA	Protection	S1P	S1PR3	Inflammation	S1PR3 worsens ischemic stroke by disrupting the blood–brain barrier via MAPK and PI3K-Akt pathways; its inhibition protects BBB integrity and improves outcomes.
Nakagawa and Aruga (2020)	Japan	Mice, Cells	IS	EC, Pericytes	Protection	S1P	ABCA1, STAT3	Inflammation	Inhibition of S1P signaling preserves BBB integrity after ischemia by suppressing STAT3 activation; probucol shows promise as a stroke treatment.
Nitzsche et al. (2021)	France	Mice, Cells	IS	EC, AS	Protection	S1P	NA	Inflammation	Endothelial S1P1 signaling protects the BBB and blood flow after stroke; selective agonists offer neuroprotection.

BBB, Blood–brain barrier; AS, Astrocytes; EC, Endothelial cell; CE, Cholesterol esters; TC, Triglycerides; FA, Fatty Acid; LDs, Lipid droplets; LPA, Lysophosphatidic acid; ROS, Reactive oxygen species; ICH, Intracerebral hemorrhagic stroke; IS, Ischemic stroke; OPCs, Oligodendrocyte progenitor cells; ACSL4, Acyl-CoA synthetase long-chain family member 4; S1P, Sphingosine-1-phosphate; S1PR3, Sphingosine-1-phosphate receptor 3.

During stroke, lipid metabolism is profoundly altered due to changes in oxygen availability, cellular stress, and inflammatory signaling (Wei et al., 2024; Adibhatla and Hatcher, 2008). One of the key lipid pathways involved is arachidonic acid metabolism, which generates pro-inflammatory mediators such as prostaglandins and leukotrienes (Wang et al., 2021; Haley et al., 2020). Following ischemic stroke, activation of COX-2 and LOXs leads to excessive production of prostaglandins and leukotrienes, which amplify microglial activation, increase cytokine release, and promote further neuronal injury (Kloska et al., 2020; Ahmad et al., 2009). In contrast, metabolites derived from omega-3 PUFAs, such as resolvins, protectins, and maresins, counteract inflammation and promote resolution by inhibiting leukocyte infiltration and reducing oxidative damage. Cholesterol metabolism also plays a key role in stroke-induced inflammation (Ament et al., 2024). ABCA1 and LXRs regulate cholesterol efflux from microglia and astrocytes, reducing pro-inflammatory lipid accumulation (Lei et al., 2025). However, stroke-induced metabolic stress downregulates ABCA1/LXR signaling, leading to cholesterol buildup, LD formation, and chronic neuroinflammation. Enhancing LXR activation has been shown to mitigate neuroinflammation and improve functional recovery post-stroke by promoting anti-inflammatory lipid mediator production (Zeng et al., 2020). In hemorrhagic stroke, lipid peroxidation is a major driver of neuroinflammation (Wu et al., 2024; Karuppagounder et al., 2018). Excessive release of free iron from blood degradation products catalyzes lipid oxidation, leading to the accumulation of ROS and toxic lipid peroxidation byproducts such as 4-HNE and MDA (Karuppagounder et al., 2018). These oxidative lipid derivatives induce neuronal apoptosis, activate inflammatory pathways, and exacerbate secondary injury. Additionally, microglia and macrophages surrounding hematomas exhibit altered lipid metabolism, shifting toward a pro-inflammatory M1 phenotype, which sustains local inflammation and hampers recovery (Wei et al., 2024; Xin et al., 2023; Pan et al., 2024).

Stroke-induced inflammation severely compromises BBB integrity, leading to increased permeability, immune cell infiltration, and cerebral edema. Lipid metabolism significantly influences BBB function through various mechanisms (Pifferi et al., 2021). Phospholipids and sphingolipids, essential components of the BBB, undergo extensive remodeling during stroke. In ischemic stroke, activation of PLA2 leads to excessive phospholipid hydrolysis, generating pro-inflammatory lipid mediators that weaken tight junction proteins (Muralikrishna Adibhatla and Hatcher, 2006). Additionally, sphingolipid metabolism, particularly S1P signaling, regulates endothelial barrier function (Fan et al., 2022). While S1P promotes endothelial stability under physiological conditions, its dysregulation post-stroke contributes to BBB breakdown and leukocyte infiltration (Fan et al., 2022). S1P receptor modulators, such as fingolimod, have shown potential in reducing BBB disruption and neuroinflammation in preclinical stroke models (Nakagawa and Aruga, 2020). Furthermore, LDs have emerged as key players in BBB regulation. Endothelial cells accumulate LDs in response to ischemic stress, potentially serving as energy reservoirs to support cellular survival (Nitzsche et al., 2021). However, excessive LDs accumulation due to impaired lipid metabolism can lead to endothelial dysfunction and heightened BBB permeability. Inflammatory cytokines, particularly TNF-α and IL-1β, further disrupt lipid homeostasis in BBB cells, exacerbating lipid oxidation and structural damage.

### 4.2 Alzheimer’s disease

Alzheimer’s disease (AD) is characterized by progressive neurodegeneration, neuroinflammation, and the accumulation of Aβ and tau pathology. Lipid metabolism is increasingly recognized as a key player in AD pathogenesis, influencing inflammatory responses and BBB integrity (Table 2). In AD, lipid metabolism disturbances contribute to amyloid-beta (Aβ) aggregation and tau hyperphosphorylation, two key pathological hallmarks of the disease (Rajmohan and Reddy, 2017). Cholesterol plays a particularly crucial role in modulating amyloid precursor protein (APP) processing, with elevated cholesterol levels promoting the amyloidogenic pathway, leading to increased Aβ production (Maulik et al., 2013). Moreover, ApoE, the primary lipid transporter in the brain, significantly affects lipid homeostasis and Aβ clearance, with the ApoE4 allele being the strongest genetic risk factor for late-onset AD (Husain et al., 2021). Oxidized lipids and lipid peroxidation products further exacerbate neuroinflammation and oxidative stress, accelerating disease progression (Ayyubova, 2024). Neuroinflammation in AD is largely driven by activated microglia and astrocytes, which respond to Aβ plaques by releasing pro-inflammatory cytokines. Lipid metabolism profoundly affects microglial activation states and their ability to clear Aβ aggregates. Triggering Receptor Expressed on TREM2 is crucial for microglial function and lipid uptake. Loss-of-function mutations in TREM2 impair microglial lipid metabolism, reducing Aβ phagocytosis and exacerbating inflammatory responses (Nugent et al., 2020). Furthermore, dysfunctional cholesterol metabolism, mediated by ApoE, particularly the ApoE4 variant, disrupts lipid homeostasis, leading to excessive LDs accumulation in microglia and astrocytes (Qi et al., 2021). This lipid overload induces oxidative stress and sustains a pro-inflammatory microglial phenotype, further contributing to synaptic dysfunction and neuronal loss. Fatty acid metabolism is another key regulator of inflammation in AD. Dysregulated omega-6/omega-3 PUFA balance leads to an overproduction of pro-inflammatory lipid mediators, such as prostaglandins and leukotrienes, which perpetuate neuroinflammation (Devassy et al., 2016; Ma et al., 2020). In contrast, bioactive lipid metabolites derived from DHA, such as resolvins and protectins, have neuroprotective and anti-inflammatory properties (Siqueira et al., 2021). However, AD patients often exhibit reduced DHA levels in the brain, contributing to a pro-inflammatory milieu. Cholesterol metabolism also modulates inflammatory responses in AD. LXRs regulate cholesterol transporters like ABCA1, facilitate lipid efflux and maintain microglial homeostasis (Zhang et al., 2015). However, in AD, LXR signaling is often impaired, leading to cholesterol accumulation, LD formation, and sustained inflammatory activation. Enhancing LXR activity has been shown to promote anti-inflammatory responses and improve lipid metabolism, making it a promising therapeutic target.

**Table 2:** Preclinical studies assessing the effect of lipids on BBB function in AD.

References	Country	Species	Involved cells	Damage or protection	Key lipids	Key factors or pathways	Effects on BBB	Mechanisms
Nugent et al. (2020)	USA	Mice	Microglia	Damage	Cholesterol	TREM2	Indirect impairment via neuroinflammation	TREM2 loss-of-function impairs microglial lipid uptake and Aβ clearance
Qi et al. (2021)	China	Cells, Human	AS, Microglia	Damage	Cholesterol, LDs	ApoE4	Lipid overload in glial cells	ApoE4 disrupts cholesterol homeostasis, inducing LD accumulation
Devassy et al. (2016)	Canada	Mice	Microglia, ECs	Protection	Omega-3	Eicosanoid synthesis	Increased permeability	Omega-3 imbalance increases pro-inflammatory lipid mediators
Siqueira et al. (2021)	Brazil	Mice	Neurons, Microglia	Protection	DHA	DHA metabolism	Anti-inflammatory milieu	Reduced DHA levels diminish resolvin/protectin production
Zhang et al. (2015)	China	Mice	Microglia, ECs	Damage	Cholesterol	LXR-ABCA1 pathway	Cholesterol buildup in BBB endothelial cells	Impaired LXR-ABCA1 signaling causes cholesterol accumulation
Kerman et al. (2022)	USA	Mice	ECs	Damage	Phospholipids	ApoE4, PLA2 pathway	Increased vascular permeability	PLA2 activation degrades phospholipids, weakening tight junctions
McManus et al. (2017)	Ireland	Mice	ECs	Protection	S1P	S1P signaling	Immune cell infiltration	Altered S1P signaling disrupts endothelial barrier function
Liu et al. (2022)	USA	Cells, Mice	ECs	Damage	Cholesterol, Phospholipids	ApoE4-mediated lipid transport	Oxidative stress, BBB breakdown	ApoE4 reduces lipid transport efficiency in BBB endothelial cells
Moulton et al. (2021)	USA	Cells, Mice	Neurons, ECs	Damage	Oxidized lipids	ApoE4, ROS pathways	Vascular leakage	Lipid peroxidation exacerbates vascular damage
Lee et al. (2017)	USA	Cells, Mice, Human	AS, ECs	Damage	LDs	Lipid storage pathways	Impaired repair mechanisms	Excessive LD storage in endothelial cells impairs BBB repair

AD, Alzheimer’s disease; Aβ, amyloid-beta; BBB, blood–brain barrier; AS, Astrocytes; ECs, Endothelial cells; TREM2, Triggering Receptor Expressed on Myeloid cells 2; ApoE, Apolipoprotein E; LDs, lipid droplets; DHA, docosahexaenoic acid; LXR, Liver X Receptor; ABCA1, ATP-binding cassette transporter A1; PLA2, Phospholipase A2; S1P, Sphingosine-1-phosphate; ROS, reactive oxygen species.

BBB dysfunction is an early pathological feature of AD, contributing to impaired Aβ clearance and increased neuroinflammation. Lipid metabolism significantly influences BBB integrity through various mechanisms, including tight junction regulation, endothelial lipid composition, and lipid-mediated signaling pathways. Phospholipid and sphingolipid metabolism play crucial roles in maintaining BBB stability. PLA2 activation in AD leads to excessive phospholipid degradation, weakening endothelial tight junctions and increasing vascular permeability (Doody et al., 2015; Kerman et al., 2022). Additionally, alterations in S1P signaling affect endothelial barrier function, facilitating immune cell infiltration and chronic inflammation. Cholesterol dysregulation also contributes to BBB breakdown in AD (McManus et al., 2017). The ApoE4 isoform is associated with reduced lipid transport efficiency, leading to cholesterol and phospholipid accumulation in BBB endothelial cells (Liu et al., 2022). This lipid imbalance disrupts endothelial function, enhances oxidative stress, and weakens BBB integrity. Furthermore, excessive lipid peroxidation, driven by ROS and toxic lipid aldehydes, exacerbates vascular damage and neuroinflammation (Moulton et al., 2021). LDs in BBB endothelial cells have recently been implicated in AD pathogenesis. Under inflammatory conditions, LD accumulation in endothelial cells can serve as an adaptive response to oxidative stress (Lee et al., 2017). However, excessive LD storage due to dysregulated lipid metabolism leads to endothelial dysfunction, impairing BBB repair mechanisms.

### 4.3 Parkinson’s disease

Parkinson’s disease (PD) is a neurodegenerative disorder characterized by dopaminergic neuronal loss, chronic neuroinflammation, and BBB dysfunction, with lipid metabolism and LDs playing critical roles in these pathological processes (Table 3). In PD, altered lipid metabolism has been linked to α-synuclein pathology, mitochondrial dysfunction, and neuroinflammation (Alecu and Bennett, 2019). Lipids such as polyunsaturated fatty acids (PUFAs) are prone to peroxidation, generating toxic lipid-derived radicals that contribute to dopaminergic neuron loss. Sphingolipid dysregulation, particularly changes in ceramide and glucosylceramide levels, has also been associated with lysosomal dysfunction and impaired autophagy, which are critical in PD pathogenesis (Mesbahi, 2023). Dysregulated lipid metabolism exacerbates neuroinflammation through the accumulation of lipid peroxidation products such as 4-HNE, which interact with α-synuclein to promote its aggregation and toxicity (Di Maio et al., 2023). Disruptions in cholesterol metabolism, particularly in the LXR/ABCA1 signaling pathway, impair lipid efflux, leading to LD accumulation in microglia and astrocytes, which sustains a pro-inflammatory state and amplifies the release of cytokines such as TNF-α, IL-1β, and IL-6, further contributing to neuronal damage (Marchi et al., 2019; Wouters et al., 2019). Although LDs initially serve as protective organelles against oxidative stress, their excessive accumulation within microglia is associated with persistent inflammation and impaired lipid clearance, exacerbating neuronal injury (Lee et al., 2017; Alarcon-Gil et al., 2022; Han et al., 2018). Similarly, BBB integrity is highly dependent on proper lipid metabolism, and its dysfunction in PD is linked to altered phospholipid and sphingolipid homeostasis (Wang et al., 2020). Overactivation of PLA2 weakens endothelial membranes and disrupts tight junctions, increasing BBB permeability and facilitating the entry of peripheral immune cells and inflammatory mediators into the brain, which in turn worsens neuroinflammation (Bate and Williams, 2015; Wu et al., 2021). Additionally, LDs within BBB endothelial cells may act as a temporary defense mechanism against oxidative damage, but prolonged lipid dysregulation contributes to endothelial dysfunction, impairing BBB repair mechanisms and further increasing barrier permeability (Wang et al., 2020). Collectively, these findings suggest that lipid metabolism and LD dynamics are central to PD pathogenesis, influencing both neuroinflammation and BBB function (Xie et al., 2020). Targeting lipid metabolic pathways, including cholesterol homeostasis, LD turnover, and phospholipid signaling, may offer novel therapeutic strategies to mitigate neuroinflammation and protect BBB integrity in PD (More et al., 2017; Takabe et al., 2016; Urano et al., 2022).

**Table 3:** Preclinical studies assessing the effect of lipids on neuroinflammation or BBB function in PD.

References	Country	Species	Involved cells	Damage or protection	Key lipids	Key factors or pathways	Effects on BBB	Mechanisms
Di Maio et al. (2023)	USA	Cells, Mice	Neuron, AS	Protection	10-NO2-OA, 4-HNE	Nrf2, α-Synuclein	Indirect neuroinflammation	10-NO2-OA products decrease α-synuclein toxicity and neuronal loss.
Marchi et al. (2019)	Italy	Cells, Human	Neuron, AS	Damage	Cholesterol, LDs	ABCA1 signaling	Lipid overload in glial cells	Impaired cholesterol efflux leads to LD accumulation, sustaining inflammation.
Wouters et al. (2019)	Belgium	Cells, Human	Neuron, AS, Microglia	Damage	Cholesterol	LXR, VCAM-1	Indirect neuroinflammation	LXR is indispensable for maintaining BBB integrity and cholesterol efflux.
Bate and Williams (2015)	UK	Cells, Mice	Neuron, AS, Microglia	Damage	Cholesterol, Phospholipids	α-Synuclein, PLA2	Increased permeability	PLA2 overactivation regulated by cholesterol, disrupting tight junctions.
Lee et al. (2017)	USA	Cells, Mice	Neuron, AS, ECs	Damage	LDs, TC	JNK/cJUN/ATF3 pathway, NF-κB pathway	Indirect neuroinflammation	LDs storage initially protects but later causes BBB dysfunction.
Wang et al. (2020)	China	Mice	ECs	Protection	Sphingolipids	Mfsd2a	Altered permeability	Disrupted sphingolipid homeostasis weakens BBB integrity.
Xie et al. (2020)	China	Cells, Mice	AS, ECs, Microglia	Protection	Omega-3, FA	NF-κB activation	Reduced neuroinflammation	DHA-derived resolvins suppress microglial activation and cytokine release.
More et al. (2017)	USA	Mice	ECs	Protection	FA	PPAR-α signaling	Enhanced BBB repair	Activated PPAR-α reduces oxidative stress and restore tight junctions.
Takabe et al. (2016)	Japan	Cells, Mice	Neurons	Damage	CEs, LDs	ACAT1 enzyme	Neuronal apoptosis	ACAT1-driven cholesterol esterification promotes neurotoxicity.

PD, Parkinson’s disease; BBB, blood–brain barrier; LDs, lipid droplets; AS, Astrocyte. 4-HNE, 4-Hydroxynonenal; 10-NO2-OA, Nitroalkene 10-nitro-oleic acid; CEs, Cholesterol esters; LXR, Liver X Receptor; ABCA1, ATP-binding cassette transporter A1; PLA2, Phospholipase A2; TC, Triglyceride; ECs, Endothelial cells; Mfsd2a, Major facilitator superfamily domain-containing 2a; COX-2, Cyclooxygenase-2; PPAR-α, Peroxisome proliferator-activated receptor alpha; ACAT1, Acyl-CoA cholesterol acyltransferase 1.

### 4.4 Other neurological disorders

Emerging research indicates that lipid metabolism and LDs play significant roles in neuroinflammation and BBB integrity across various neurodegenerative diseases, including multiple sclerosis (MS), Huntington’s disease (HD), and amyotrophic lateral sclerosis (ALS) (Table 4). In MS, disturbances in lipid metabolism have been linked to disease progression (De Nuccio et al., 2011). Lipid metabolism is essential for myelin synthesis and maintenance, and disruptions in lipid homeostasis contribute to demyelination and neuroinflammation (López-Muguruza and Matute, 2023). Oligodendrocytes rely on lipid biosynthesis to generate myelin sheaths, and their dysfunction leads to impaired remyelination in MS lesions (López-Muguruza and Matute, 2023). Moreover, lipid mediators such as eicosanoids and sphingolipids play pivotal roles in immune cell activation and neuroinflammation, influencing disease progression (Podbielska et al., 2021). Notably, increased levels of cholesteryl esters have been observed in MS patients, suggesting a potential role in the disease’s pathology. These alterations in lipid composition may influence inflammatory processes and impact BBB integrity, thereby contributing to the neuroinflammatory environment characteristic of MS (Lorincz et al., 2024; Fellows et al., 2015). In HD, lipid abnormalities have been observed in both the CNS and peripheral tissues, with changes in cholesterol homeostasis affecting synaptic function and neuronal viability (Leoni and Caccia, 2015). The mutant huntingtin protein disrupts lipid metabolism by impairing SREBPs, leading to reduced cholesterol biosynthesis and altered phospholipid composition, which may exacerbate neuronal dysfunction and degeneration (González-Guevara et al., 2020). HD is also associated with significant lipid metabolic disturbances. Elevated cholesteryl ester levels have been detected in specific brain regions, such as the caudate and putamen, of HD patients (Phillips et al., 2020). This accumulation may affect neuronal function and viability, potentially exacerbating neuroinflammation and compromising BBB integrity (Joshi et al., 2019). The presence of increased cholesteryl esters underscores the importance of lipid metabolism in HD pathology. In ALS, lipid metabolism alterations have been implicated in motor neuron degeneration, energy metabolism deficits, and neuroinflammation (D'Amico et al., 2021). ALS patients often exhibit hypermetabolism and dyslipidemia, with increased lipid oxidation and altered fatty acid composition in motor neurons (D'Amico et al., 2021). Dysfunctional LDs in astrocytes and microglia also contribute to oxidative stress and inflammatory responses, aggravating disease pathology. While direct evidence linking LDs and lipid metabolism to neuroinflammation and BBB dysfunction in ALS is less extensive, metabolic alterations have been observed in ALS patients (Díaz et al., 2024; Mesev et al., 2017). These metabolic changes could influence lipid processing and storage, potentially affecting inflammatory responses and BBB function. Further research is necessary to elucidate the specific roles of lipid metabolism and LDs in ALS pathology.

**Table 4:** Preclinical studies assessing the effect of lipids on neuroinflammation or BBB function in other neurological disorders.

References	Country	Species	Involved cells	Function	Key lipids	Disease	Key factors	Effects on BBB	Mechanisms
De Nuccio et al. (2011)	Italy	Mice	Oligodendrocytes, Microglia	Damage	CEs	MS	PPAR-γ signaling	Increased permeability	Cholesteryl ester accumulation drives demyelination.
Lorincz et al. (2024)	Czech	Human	ECs	Protection	HDL-C	MS	Cholesterol metabolism	Reduced immune infiltration	NA
Fellows et al. (2015)	USA	Human	ECs	Protection	HDL-C	MS	CD80+, CD19+	Reduced neuroinflammation	Maintaining BBB integrity following the first demyelinating event
Phillips et al. (2020)	Australia	Human	Neurons	Damage	CEs	HD	ACAT1 enzyme	Cholesterol metabolism	ACAT1-driven cholesterol esterification in striatum exacerbates neurodegeneration.
Joshi et al. (2019)	USA	Cells, Mice, Human	Neurons, Microglia	Damage	LDs	HD	NLRP3 inflammasome	Pro-inflammatory polarization	LDs in microglia activate NLRP3, worsening neuroinflammation.
Díaz et al. (2024)	Spain	Human	Neurons	Damage	Phospholipids, CEs	ALS	PLA2 pathway	Mitochondrial dysfunction	PLA2-mediated phospholipid breakdown impairs mitochondrial integrity.
Mesev et al. (2017)	USA	mice	ECs	Damage	Sphingolipids	ALS	Ceramide synthase, COX-2	BBB leakage	Sphingolipid imbalance disrupts endothelial tight junctions.

MS, Multiple sclerosis; HD, Huntington’s disease; ALS, Amyotrophic lateral sclerosis; CEs, Cholesteryl esters; ECs, Endothelial cells; BBB, Blood–brain barrier; LDs, Lipid droplets; PPAR-γ, Peroxisome proliferator-activated receptor gamma; HDL-C, high-density lipoprotein cholesterol; ACAT1, Acyl-CoA cholesterol acyltransferase 1; NLRP3, NOD-, LRR- and pyrin domain-containing protein 3; PLA2, Phospholipase A2.

## 5 Applications in neurological disorders’ therapy

### 5.1 Regulating lipid metabolism to improve neurological disorders

Lipid metabolism, LDs, and liposomes have emerged as significant therapeutic targets in the treatment of neurological disorders (Gigliobianco et al., 2019; Mondal and Ghosh, 2023). Modulating lipid homeostasis and LD dynamics offers promising strategies for alleviating neuroinflammation, supporting neuronal survival, and restoring brain homeostasis in conditions such as AD, PD, and MS (Alecu and Bennett, 2019; Chiurchiù et al., 2022; Kang and Rivest, 2012). For instance, enhancing the activity of enzymes involved in lipid biosynthesis, such as LXRs and ABCA1, may promote cholesterol efflux, reduce lipid accumulation in glial cells, and thereby alleviate neuroinflammation (Kang and Rivest, 2012). Additionally, targeting lipid signaling pathways, such as the SREBPs and TREM2, may help control LD formation and mitigate chronic inflammatory responses in microglia and astrocytes, two critical cell types involved in neuroinflammation (Wang et al., 2025; Loving and Bruce, 2020). LDs, as intracellular lipid storage organelles, play a pivotal role in cellular energy balance and inflammation regulation. Excessive LD accumulation and lipid peroxidation are characteristic features of several neurodegenerative diseases, and regulating LD metabolism may help prevent oxidative stress and chronic neuroinflammation (Yin, 2023). Particularly in diseases like AD, microglial LD accumulation has been linked to prolonged inflammatory states and neuronal damage. Pharmacological interventions targeting LD-associated proteins, such as perilipins, may provide novel therapeutic opportunities by limiting inflammation and promoting tissue repair (Mi et al., 2023; Simpson and Oliver, 2020; Vigouroux et al., 2011).

### 5.2 Lipid particles for drug delivery across the blood–brain barrier

Liposomes, as lipid-based nanocarriers, are increasingly being explored as drug delivery systems for neurological disorders (Vieira and Gamarra, 2016). Their ability to encapsulate hydrophobic drugs, protect them from degradation, and facilitate targeted delivery across the BBB makes them ideal candidates for treating brain diseases (Table 5). Liposomes can be designed to encapsulate anti-inflammatory agents, neuroprotective compounds, or gene therapies, providing sustained and localized treatment in the brain (Wu et al., 2023; Cascione et al., 2020; Kahana et al., 2021; Nong et al., 2024; Al-Ahmady et al., 2019; Lee et al., 2014; Yang et al., 2018; Ediriweera et al., 2025). Notably, liposomal formulations of compounds such as curcumin and cannabinoids have demonstrated anti-inflammatory and neuroprotective potential in models of AD and PD (Alecu and Bennett, 2019; Pandian et al., 2022; Zhang et al., 2018). Additionally, liposomes can be engineered to target specific cell types within the brain, such as neurons, astrocytes, and microglia, enabling tailored therapeutic approaches (Kahana et al., 2021; Pandian et al., 2022; Moreira et al., 2024; Sela et al., 2023). Liposomes can also deliver lipid-lowering agents or molecules that modulate lipid metabolism to restore proper lipid homeostasis in the brain, potentially alleviating neuroinflammation and improving cognitive function (Torres et al., 2021; Campos-Peña et al., 2022).

**Table 5:** Liposome-based nano drugs for CNS diseases and their status.

References	Country	Disease	Product name	Species	Route	Lipid resources	Delivered drug	Mechanism
Liu et al. (2021)	China	AD	Tf-HA-Cur	Mice	IV	Liposomes	Curcumin	Improving the cognitive and learning ability.
Mourtas et al. (2014)	Greece	AD	Lipid-PEG-curcumin	Human	IV	Liposomes	Curcumin	Inhibition of amyloid-β aggregation
Zhang et al. (2018)	China	PD	CPC-NPs	Mice	IV	Liposomes	Curcumin	Inhibition of amyloid-β aggregation
Kahana et al. (2021)	Portugal	PD	Liposome-DA	Mice	IP	Liposomes	Dopamine	Enhances DA penetration
Sela et al. (2023)	Israel	PD	BTL-SynO4	Mice	IV	Liposomes	mAbs	Enhance neuronal activity
Nong et al. (2024)	Israel	IS	NC-VCAM	Mice	IV	LCs	VCAM1	Reduce neuroinflammation
Al-Ahmady et al. (2019)	UK	IS	Liposomes	Mice	IV	Liposomes	NA	Blocking inflammation, neuronal repair
Lee et al. (2014)	Germany	MS	PEG-MP	Mice	IV	PEG	MP	Reduces infiltration of T cells and macrophages/microglia
Yang et al. (2018)	USA	ALS	Cocktail liposomes	Cells	Incubation	Liposomes	Riluzole	Enhance BBB permeability
Ediriweera et al. (2025)	Australia	ALS	ASO-loaded nanoparticle	Mice	IV	CaP-NPs	Tofersen	Reducing misfolded proteins in motor neurons

CNS, central nervous system; AD, Alzheimer’s disease; PD, Parkinson’s disease; MS, multiple sclerosis; HD, Huntington’s disease; Aβ, amyloid-beta; IV, Intravenous; IP, Intraperitoneal; LD, Lipid droplet; PEG, polyethylene glycol; CPC-NPs, curcumin-loaded polysorbate 80-modified cerasome nanoparticles; mAbs, Monoclonal antibodies; IS, Ischemic stroke; LCs, Lipid nanocarriers; VCAM1, vascular cellular adhesion molecule-1; MP, Methylprednisolone; ASOs, Antisense oligonucleotides; CaP-NPs, calcium phosphate lipid nanoparticles.

## 6 Conclusions and prospects

Lipid metabolism and LDs play central roles in regulating neuroinflammation and maintaining BBB integrity in neurological disorders. Dysregulation of lipid homeostasis contributes to chronic inflammation, neuronal damage, and BBB dysfunction, exacerbating diseases like stroke, AD, PD, and MS. Targeting lipid metabolic pathways, modulating LD dynamics, and utilizing lipid-based nanocarriers like liposomes offer promising therapeutic strategies to mitigate inflammation, restore lipid balance, and improve drug delivery to the brain. Future research should focus on developing specific interventions that regulate lipid metabolism and LD formation, offering new avenues for treating neurodegenerative diseases and enhancing therapeutic efficacy.

## Glossary

Aβ: Amyloid-beta
ABCA1: ATP-binding cassette transporter A1
ABCG1: ATP-binding cassette transporter G1
ACAT1: Acyl-CoA cholesterol acyltransferase 1
ACSL4: Acyl-CoA synthetase long-chain family member 4
AD: Alzheimer’s Disease
ALS: Amyotrophic Lateral Sclerosis
ApoE: Apolipoprotein E
APP: Amyloid Precursor Protein
AS: Astrocytes
BBB: Blood–Brain Barrier
CEs: Cholesteryl Esters
CNS: Central Nervous System
COX: Cyclooxygenase
COX-2: Cyclooxygenase-2
DHA: Docosahexaenoic Acid
EC: Endothelial Cell
ER: Endoplasmic Reticulum
FA: Fatty Acid
HDL-C: High-Density Lipoprotein Cholesterol
HD: Huntington’s Disease
ICH: Intracerebral Hemorrhagic Stroke
IFN-γ: Interferon-gamma
IL-1β: Interleukin-1 beta
IL-6: Interleukin-6
IR: Insulin Receptor
IS: Ischemic Stroke
LDs: Lipid Droplets
LDLR: Low-Density Lipoprotein Receptor
LOX: Lipoxygenase
LPA: Lysophosphatidic Acid
LXRs: Liver X Receptors
MDA: Malondialdehyde
MD: Medical Doctor
MFSD2A: Major Facilitator Superfamily Domain-containing 2A
MS: Multiple Sclerosis
NF-κB: Nuclear Factor Kappa-Light-Chain-Enhancer of Activated B Cells
PD: Parkinson’s Disease
PEGylation: Polyethylene Glycol Modification
PLA2: Phospholipase A2
PLINs: Perilipins
PPAR-γ: Peroxisome Proliferator-Activated Receptor Gamma
PUFA: Polyunsaturated Fatty Acid
ROS: Reactive Oxygen Species
S1P: Sphingosine-1-phosphate
S1PR: Sphingosine-1-phosphate Receptor
SREBPs: Sterol Regulatory Element-Binding Proteins
TC: Triglycerides
TfR: Transferrin Receptor
TG: Triglycerides
TNF-α: Tumor Necrosis Factor-alpha
TREM2: Triggering Receptor Expressed on Myeloid cells 2
4-HNE: 4-Hydroxynonenal
10-NO_2_-OA: 10-Nitro-oleic Acid
